# Rape of the Red Back

**Published:** 1899-09

**Authors:** 


RAPE OF THE RED BACK.—Keasbey & Mattison, of Ambler, Pa., manufacturers of a proprietary medicine called "Alkalithia," have sent out a pamphlet alleged to be a reprint from the TEXAS MEDICAL JOURNAL. The JOURNAL repudiates it, and denounces such method as disreputable. No such paper ever appeared in this journal. In the March number (1896) of the JOURNAL there appeared an excellent paper on the "Cause and Treatment of the Uric Acid Diathesis," by Dr. N. A. Olive, of Waco, together with a part of the discussion which followed its reading before the Central Texas Medical Society, January 14, 1896. Dr. J. W. Hunter, the traveling agent for Keasbey & Mattison, was present and participated in the discussion. The JOURNAL published the discussion, including a part of Dr. Hunter's remarks, but rejected what he had to say in laudation of the proprietary medicine which he was selling, and inserted at that point the following: "Balance of Dr. Hunter's remarks omitted, as they were mainly in advocacy of a proprietary article in which he is interested.—ED."

This enterprising firm, Keasbey & Mattison, now reproduce the paper of Dr. Olive, with the discussion as it appeared in the JOURNAL, and have added all that stuff which we had blue pencilled and thrown out, Dr. Hunter's eulogy on alkalithia and some "testimonials," and have sent it out as "revised reprint from the TEXAS MEDICAL JOURNAL." We decidedly object to the JOURNAL's yielding up its virtue to such forcible methods. It is forcing an endorsement, and is unbecoming any reputable concern.

